# [Retracted] Study of miR-143 expression in stomach cancer

**DOI:** 10.3892/ol.2023.13743

**Published:** 2023-03-06

**Authors:** Shao-Jun Huang, Yu-Fang Zhu, Zeng Liu, Qing-Feng Li, Zhao-Yuan Li, Wen-Rong Fu

Oncol Lett 16: 4367–4371, 2018; DOI: 10.3892/ol.2018.9173

Following the publication of this paper, it was drawn to the Editor's attention by a concerned reader that the flow cytometric data in Fig. 3A on p. 4370, comparing both between and within the ‘Control’ and ‘Overexpression’ experimental panels, exhibited unexpected repeating patterns with respect to the distribution of the data. The authors were asked to provide an explanation to account for the potentially anomalous appearance of the data in this figure, although no reply was received from them within the requested time period for a response. The Editor of *Oncology Letters* has therefore decided that this paper should be retracted from the Journal on the grounds of a lack of confidence in the presented data. The Editor apologizes to the readership for any inconvenience caused.

